# Platelet activating factor-induced mast cell degranulation is inhibited by rupatadine, and to a lower extent by levocetirizine and desoratadine, in a mast cell line (LAD-2)

**DOI:** 10.1186/2045-7022-3-S2-O6

**Published:** 2013-07-16

**Authors:** Joaquim Mullol, Rosa Muñoz-Cano, I Torres-Atencio, E Ainsua, M Martin, J Sanchez-Lopez, Joan Bartra, Cesar Picado, Antonio Valero

**Affiliations:** 1 Hosp Clinic, IDIBAPS, Barcelona, Spain

## Background

Platelet activating factor (PAF) is a lipid mediator that appears to be involved in the pathophysiology of several allergic reactions such as anaphylaxis and potentially urticaria and allergic rhinitis. The role of rupatadine, a drug with dual antihistamine and anti-PAF effect, in mast cell (MC) degranulation is not known. The objective of this study was to investigate the expression of PAF receptors and the effect of rupatadine on PAF-induced MC degranulation compared with other second generation antihistamines (desloratadine, levocetizine) and a pure specific PAF inhibitor in a human mast cell line (LAD-2).

## Methodology

MC degranulation was evaluated by the â-hexosaminidase and histamine release while PAF receptor expression was evaluated by western blot. After stimulation with PAF in a dose-response and time course manner, the optimal PAF conditions to induce LAD-2 degranulation were identified (10 µM and 30 minutes). The effects of rupatadine, desloratadine, and levocetirizine (from 1µM to 100 µM) on PAF-induced LAD-2 degranulation were investigated. The inhibitory effect of CV6209 (specific anti-PAF) at 2 µM was used as positive control in all experiments.

## Results

Protein expression of the PAF receptor was found in LAD-2 cells. Rupatadine (5 to 10 µM, p<0.005) and levocetirizine (5 µM, p<0.01) but not desloratadine inhibited PAF-induced â-hexosaminidase release. Rupatadine (1 to 10 µM, p<0.01), levocetirizine (1 to 25 µM, p<0.05), and desloratadine (10 µM, p<0.05) also inhibited PAF-induced histamine release.

## Conclusions

This study shows that the ant-H1 compunds rupatadine, and to a lower extent levocetirizine and desloratadine, have an anti-PAF effect in the mast cell line LAD-2, suggesting that rupatadine could be more effective than other antihistamine drugs in those allergic disorders where PAF may act as an important inflammatory mediator.

